# Alignment-free clustering of transcription factor binding motifs using a genetic-k-medoids approach

**DOI:** 10.1186/s12859-015-0450-2

**Published:** 2015-01-28

**Authors:** Pilib Ó Broin, Terry J Smith, Aaron AJ Golden

**Affiliations:** 1 Department of Genetics, Albert Einstein College of Medicine, 1301 Morris Park Avenue, Bronx, New York, 10461 USA; 2 National Centre for Biomedical Engineering Science, National University of Ireland, University Road, Galway, Ireland; 3 Department of Mathematical Sciences, Yeshiva University, New York, 10033 NY USA

**Keywords:** Transcription factor, Motif, Clustering, Genetic algorithm

## Abstract

**Background:**

Familial binding profiles (FBPs) represent the average binding specificity for a group of structurally related DNA-binding proteins. The construction of such profiles allows the classification of novel motifs based on similarity to known families, can help to reduce redundancy in motif databases and *de novo* prediction algorithms, and can provide valuable insights into the evolution of binding sites. Many current approaches to automated motif clustering rely on progressive tree-based techniques, and can suffer from so-called frozen sub-alignments, where motifs which are clustered early on in the process remain ‘locked’ in place despite the potential for better placement at a later stage. In order to avoid this scenario, we have developed a genetic-*k*-medoids approach which allows motifs to move freely between clusters at any point in the clustering process.

**Results:**

We demonstrate the performance of our algorithm, GMACS, on multiple benchmark motif datasets, comparing results obtained with current leading approaches. The first dataset includes 355 position weight matrices from the TRANSFAC database and indicates that the *k*-mer frequency vector approach used in GMACS outperforms other motif comparison techniques. We then cluster a set of 79 motifs from the JASPAR database previously used in several motif clustering studies and demonstrate that GMACS can produce a higher number of structurally homogeneous clusters than other methods without the need for a large number of singletons. Finally, we show the robustness of our algorithm to noise on multiple synthetic datasets consisting of known motifs convolved with varying degrees of noise.

**Conclusions:**

Our proposed algorithm is generally applicable to any DNA or protein motifs, can produce highly stable and biologically meaningful clusters, and, by avoiding the problem of frozen sub-alignments, can provide improved results when compared with existing techniques on benchmark datasets.

**Electronic supplementary material:**

The online version of this article (doi:10.1186/s12859-015-0450-2) contains supplementary material, which is available to authorized users.

## Background

Transcription factors (TFs) are an important group of DNA-binding proteins whose interaction with their cognate sequence-specific binding sites results in the regulation of gene expression. These transcription factor binding sites (TFBSs) are short, degenerate sequences, usually in the order of 6-32bp in length [1], and are most commonly represented in the form of a position specific scoring matrix (PSSM), or position weight matrix (PWM). A PSSM is a 4 x *ℓ* matrix created from the alignment of known binding sites, where *ℓ* is the motif length, and each matrix entry, *f*_*bi*_, represents the probability of observing nucleotide *b* in position *i* of the motif [2].

**Figure 1:**
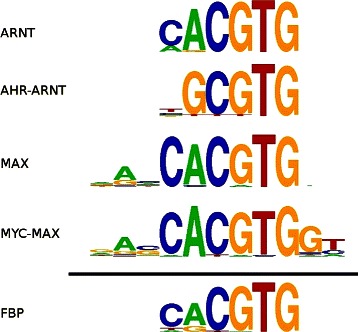
**Sample familial binding profile.** Sample FBP for four transcription factors from the basic Helix-Loop-Helix structural family. Columns which have low information content or are only present in a small number of the individual motifs are typically excluded from the FBP.

The concept of a familial binding profile (FBP), or average binding specificity for a group of structurally related TFs (as shown in Figure 1), was introduced by the authors in [3], when they manually constructed 11 FBPs from 71 non-zinc-finger motifs taken from the JASPAR database [4]. FBPs are an important tool in regulatory genomics and serve a multitude of purposes: i) they can be used as informative priors for motif discovery algorithms, either biasing the search to TFs from a particular structural family, or providing a way to filter out spurious patterns and thereby increasing sensitivity [3,5], ii) they can be used to classify novel binding proteins based on their similarity to the binding affinities of known structural families [6,7], iii) they can be used to reduce redundancy in motif databases where minor variations or submotifs from the same binding site are incorrectly labelled as separate motifs; this redundancy reduction can also be applied to motif finding algorithms, either to merge similar motif predictions from a single algorithm or to combine results from multiple algorithms [8,9], and iv) they can be used to analyze binding site turnover and provide insights into how DNA-binding mechanisms have evolved over time [10].

While [3] described the manual creation of FBPs, there have since been numerous studies which have examined various metrics for motif comparison as well as methods for their automated clustering.

Determination of motif similarity can be broadly classified into two approaches: alignment-based methods, which implement a column-by-column scoring approach for each alignment provided by sliding motifs against one another in both forward and reverse directions, and alignment-free methods, which compare motifs directly based on shared features. Column scoring for alignment-based techniques can be based on metrics such as sum squared distance (SSD), Pearson’s correlation coefficient (PCC), and average Kullback-Leibler (AKL) distance, many of which have previously been examined in detail [9,10]. A method for alignment-free motif comparison was provided by the MoSta tool [11], which uses the asymptotic covariance of overlapping word sets between two motifs on a random sequence to determine similarity, with more similar motifs showing a higher degree of overlap. Another approach is taken by the authors in [12], who first convert each PSSM to a *k*-mer frequency vector (KFV), a 4^*k*^-dimensional vector comprising the frequency of each possible *k*-mer in a given motif, and then determine similarity using metrics such as PCC, Euclidean distance, or cosine distance. They demonstrate that the KFV approach outperforms alignment-based techniques in motif retrieval experiments designed to identify optimal motif similarity measures.

Currently, one of the most popular tools for motif clustering is the STAMP platform [10,13]. It offers a choice of column comparison metrics and performs pairwise gapped or ungapped local [14], or global [15] alignment, with progressive multiple alignment being performed using a UPGMA [16] guide tree. A known problem associated this type of agglomerative approach is that it can suffer from so-called frozen subalignments [17], where a datum seemingly well-clustered early on in the tree building process is later found to better match another cluster. STAMP therefore also provides an option for iterative refinement, although this can take much longer given that each motif from the initially constructed tree must be removed and realigned to the remaining motifs. MoSta, on the other hand provides its own non-tree-based clustering approach, which includes a threshold designed to ensure that the FBP resulting from successive merges maintains a high degree of similarity to each of its contributing members. This threshold helps to maintain structural homogeneity (defined as the proportion of cluster members which share the same structural class), an important concept in creating biologically meaningful FBPs.

Here, we are interested in an approach which would i) make use of the demonstrated success of the alignment-free KFV approach, and ii) allow motifs to move freely between clusters at any stage in the clustering process, thereby reducing the likelihood of convergence to a local rather than a global minimum, and obviating the need for post-clustering iterative refinement. Combining KFV calculation with a partitional clustering technique such as *k*-means [18] would provide such an approach, *k*-means however, is known to be highly sensitive to the effects of outliers. We therefore explore the use of the *k*-medoids algorithm [19] which, rather than calculating a group mean, uses the cluster member with the smallest total pairwise distance to all other cluster members as the group representative instead. This not only has the advantage of being resistant to outlier effects, but also provides the additional benefit of not having to repeatedly calculate a multiple alignment for each cluster as would a *k*-means approach. The *k*-medoids algorithm does however have two major associated problems of its own: i) like *k*-means, it can be sensitive to initial conditions, converging on different solutions depending on the randomly chosen starting medoids, ii) it performs a local search only, providing solutions exclusively for the value of *k* provided; ideally our approach should automatically determine the optimal number of clusters for any dataset provided. To address these two issues, we propose the use of a genetic algorithm (GA).

**Figure 2:**
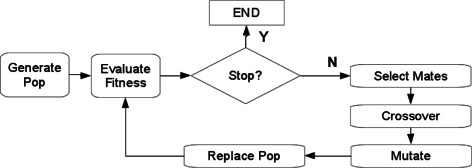
**Genetic algorithm overview.** After the population is initialized, the algorithm iterates between evaluating the solutions from the current population and generating new solutions from selected candidates through various evolutionary operators. Termination usually occurs after a specified number of iterations (‘generations’) have elapsed, when an acceptable solution (such as within an allowable error threshold) has been found, or, after a fixed period during which the fitness has remained relatively constant.

Genetic algorithms, based on early work by Fraser [20] and later popularized by Holland [21] and Goldberg [22], are a stochastic optimization technique making use of a population of candidate solutions. These candidate solutions, commonly encoded as binary strings (although representation as integers and floating-point numbers are also popular), are iteratively evaluated for their effectiveness, or ‘fitness’, for a given problem domain, which is often termed a ‘fitness landscape’ or ‘search space’. The solutions and then combined through the use of evolutionarily inspired genetic operators such as selection, mutation, and crossover, to form the next generation of candidates (Figure 2), with each successive generation providing increasingly ‘fit’ solutions. The parallel search capabilities of GAs (simultaneous sampling of multiple points in the fitness landscape) coupled with their ability to potentially ‘escape’ local minima through the introduction of ‘novelty’ via mutation, make them ideally suited to complex, noisy problem domains, and their use for both multiple sequence alignment [23,24] and motif discovery [25,26] has been well established. By embedding the *k*-medoids algorithm within a GA framework and choosing a suitable ‘fitness function’ to evaluate candidates, we can leverage the local search capabilities of the *k*-medoids algorithm while using the GA to both perform global search for the optimal number of clusters, and to provide multiple initializations for the *k*-medoids algorithm, reducing the potential impact of poorly chosen starting medoids. Below, we detail the implementation and performance of such an alignment-free, non-tree-based approach to the motif clustering problem based on a software tool we have developed called GMACS.

## Implementation

In this section we provide details on the implementation of our genetic-*k*-medoids approach, GMACS. We describe the algorithm in terms of three stages. The first of these is the initialization stage where we construct a distance matrix for the motifs in our dataset based on *k*-mer frequencies and create an initial random population of candidate clustering solutions. The second stage details the process of assigning a ‘fitness’ score to each solution in the population through the use of one iteration of the *k*-medoids algorithm and the silhouette metric. The third step details the evolutionary process, the way in which the ‘fitter’ solutions are selected and recombined during each ‘generation’, or iteration of the genetic algorithm.

### Initialization

The first step in our algorithm is to create a matrix of pairwise distances between the weight matrices in our dataset. We begin by calculating the information content for each motif position, *i*, as follows:

(1) $$ I_{i}=2+\sum_{j}p_{ij}\cdot {log}_{2}\left(p_{ij}\right)  $$

where *p*_*ij*_ is the probability of observing nucleotide *j* in position *i* of the motif. We then trim the motifs based on a user-defined threshold (default 0.3) to remove less informative edge columns. While trimming, we ensure that motifs are not shortened below a minimum core length of four nucleotides. Next, we create *k*-mer frequency vectors for each of the trimmed motifs. A value of four was chosen for *k* based on the results reported in [12], where the authors explored various combinations of *k*-values and distance metrics. The choice of *k*=4 is also congruent with the fact that tetranucleotide frequencies have previously been shown to convey considerable genomic information [27]. Each element in a KFV represents the frequency of a particular *k*-mer (‘AAAA’, ‘AAAC’, ‘AAAG’, and so on), resulting in a vector of length 4^*k*^. Once we have created our KFVs, we use the cosine distance to populate our distance matrix, where the distance between two motifs, *a* and *b* is defined as:

(2) $$ d_{cos}(a,b)=1-\frac{a \cdot b}{\|a\|\|b\|}  $$

and motifs with a *d*_*cos*_ close to zero are regarded as highly similar. In contrast to an agglomerative approach, these pairwise distances remain unchanged and do not need to be re-calculated as the clustering progresses and motifs are merged.

### Fitness calculation

Each candidate solution in the GA population encodes *K*, the number of clusters in the solution, and a vector *m*, of length *K*, which indicates the medoid for each of these clusters. During initialization, the value of *K* for each solution is randomly chosen in the range {2…*n*−1}, where *n* is the number of motifs in the input dataset. This range is used as the *K*=1 solution provides no real benefit since all motifs are clustered together regardless of similarity, while conversely, the *K*=*n* solution places each motif in its own singleton cluster and is of little use for reducing redundancy. Once the number of clusters for each solution has been established, starting medoids are also randomly chosen. When all of the candidate solutions are initialized, we proceed to calculate the fitness for each member of the population.



To calculate the fitness for a candidate solution, we first perform one round of the *k*-medoids algorithm as outlined in Algorithm 1. Briefly, we first assign each motif to its nearest medoid based on the distance matrix and then calculate the overall cost of the cluster configuration, defined as the total distance of each motif to its nearest medoid. Then, for each of the currently selected medoids, *i*, we swap *i* with a non-medoid motif, *j*, and recalculate the overall cost. If the new cost is lower than the previous cost, we keep the new medoid. If medoids are updated during the swap step, the assignment step is repeated. This local search step will choose ‘good’ medoids based on the current value of *K*, and greatly speeds up the convergence of the GA towards promising solutions. Carrying out only one round of the *k*-medoids algorithm provides us with the benefit of improved current solutions through local search, without the computational overhead of a full *k*-medoids approach, which typically runs until no further updates to the medoids can be made to lower the total cost of the cluster configuration. As the goal of our GA is to both determine the optimal number of clusters and their membership, the fitness function will necessarily also include some measure of how well the data are clustered. Two methods commonly used are the Gap statistic [28] which calculates the difference between successive values of *K* for the test data and a bootstrapped reference dataset, and the CH-metric [29], which provides a ratio of intra- and inter-cluster distance. The authors in [10] also found that, for the tree-based approach, a log-based equivalent of the CH-metric (*C* *H*_*log*_) was preferable to the standard metric – this log-based version was also used by [12]. Here however, we use the Silhouette metric, which has been shown to be well-suited to partitional approaches [30] and is defined as follows:

(3) $$ s(i)=\frac{b(i)-a(i)}{\text{max}\{a(i),b(i)\}}  $$

In this metric, *a*(*i*) is the average dissimilarity of motif *i* to all other motifs in its own cluster, and *b*(*i*), is the average dissimilarity of motif *i* to all motifs in its nearest neighbouring cluster; *s*(*i*) is therefore an indication of whether or not an individual motif is well-placed in the clustering, or if it would be clustered more appropriately elsewhere. By calculating a silhouette score each motif in the dataset, we generate an overall measure of cluster quality.

### Evolutionary process

Once fitness values have been assigned to each solution, they are ranked in preparation for the evolutionary process. GMACS implements a linear ranking system incorporating a selective pressure parameter which can be used to adjust the strength of the selective bias towards fitter individuals. Linear ranking is commonly used as opposed to direct fitness values in order to avoid situations where a small number of disproportionately successful solutions leads to the premature convergence of the population. We follow an incremental or steady-state GA (SSGA) replace-worst strategy, such that, in each generation, the bottom 5% of the parent population will be replaced by newly-created offspring. This represents a more gradual progression towards fitter solutions in contrast to a more aggressive generational strategy where the entire population is replaced at each iteration and relatively fit solutions may be lost over time due to the stochastic nature of the algorithm.

We use roulette wheel, or fitness proportionate selection, to choose the two parent solutions when generating offspring for replacement. In this form of selection, each individual in the population is assigned a ‘slice’ of an imaginary roulette wheel which is proportionate to its fitness within the context of the current population. The wheel is ‘spun’, and solutions or individuals which have larger slices of the wheel will have a greater probability of being selected for recombination. Weaker solutions will still have a small probability of selection, and this is in keeping with the fundamental theory of genetic algorithms, namely that part of a less fit solution’s genotype may still be beneficial at a later stage when combined with genes from another solution.

**Figure 3:**
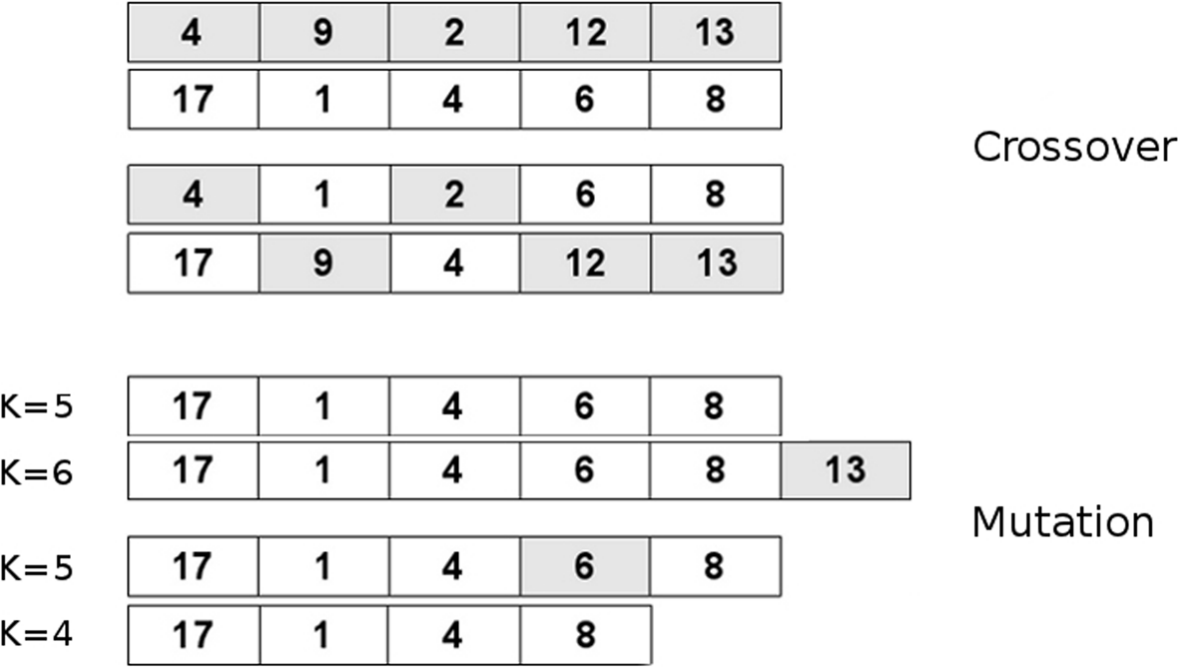
**Solution representation and evolutionary operators.** Depiction of crossover and mutation in GMACS. The upper section shows the medoid vector representation of two selected parents, one wholly-shaded and one wholly-unshaded. Shown below them are the two offspring resulting from their uniform crossover. The lower section demonstrates the two modes of mutation: addition or removal of a randomly selected cluster, shown as shaded.

The medoid vector representation and the effects of the crossover and mutation operators on those encodings are shown in Figure 3. Two parents are shown at the top of the figure, one shaded and one unshaded. Both have five clusters (a point we will return to shortly), and the index of the motif currently assigned as the medoid for each of these clusters is shown as an integer value. The form of crossover we use is termed ‘uniform crossover’, meaning that each separate gene in an offspring’s genotype has an equal chance of coming from either parent. This type of crossover, while less common than single- or multi-point crossover, arguably produces a wider range of genotypes, exploring more of the search space. In order to explore solutions with different numbers of clusters (particularly those which may not arise as part of the random initialization), the mutation operator functions by perturbing the *K*-value for a given solution, either adding a cluster by copying the existing medoids and choosing at random an additional medoid from the remaining motifs, or removing a cluster (provided *K*>2), by randomly choosing a medoid to delete. The probability of a mutation occurring is typically kept quite low (lest the GA risk becoming a totally random walk), and here, the rate is set at 0.05.

**Figure 4:**
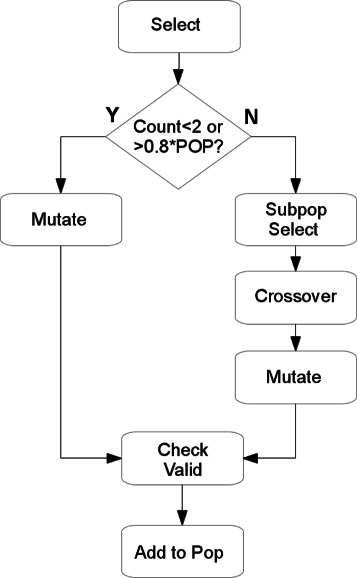
**Modified selection process.** This modified selection process is designed to only allow the recombination of individuals sharing the same *K*-value. In cases where no suitable mate exists, or where the pre-determined diversity threshold has been exceed, mutation will occur (with the standard probability) without crossover.

As individuals in the population may have different *K*-values, special consideration must be given when carrying out the selection step. During the *k*-medoids phase of the fitness calculation, the current set of medoids is updated to a partially-optimized state. Crossover of medoids between solutions containing different numbers of clusters would result in a disruption to this improvement. If the algorithm were carrying out a full *k*-medoids implementation this would not present a problem since the medoids would be optimized on the next pass of the fitness function. Since however, only one pass through the medoids occur, crossover is constrained to individual sharing the same number of clusters. Figure 4 shows the modified selection process to account for this fact. Once the first parent is selected, a check is made to see if there are any other individuals in the population with the same number of clusters – if there are, then the second mate is selected from within that subpopulation and crossover occurs as normal. If the individual, however, is the only member of the population with that specific value of *K*, then no valid mate exists and the crossover step is skipped. Figure 4 also shows two additional features of the algorithm design. The first of these is the concept of population diversity, expressed as the proportion of the population with the same *K*-value. When this value is greater than a pre-defined threshold (default: 0.8), it gives an indication that the population has largely converged on a solution with a specific number of clusters and mutation is temporarily increased to both maintain the remaining diversity and encourage further exploration of cluster space. The second feature is the offspring validity check which is necessary after crossover and/or mutation to ensure that there are no duplicate medoids as a result of the recombination or mutation.

## Results

### Motif comparison

Our first dataset consists of 355 motifs from the six largest structural families in the TRANSFAC [31] database and has previously been used by [7,11,12], and [10] to benchmark retrieval accuracy. We have previously described MoSta and STAMP, the remaining approach against which we compare GMACS is based on work by the authors of [7] in which they construct a multi-class motif classifier with feature selection by applying sparse multinomial logistic regression (SMLR) to feature vectors of length 1390 which incorporate measures such as nucleotide frequencies, presence of palindromic features, and previously published submotifs. The retrieval accuracy for each metric is a measure of its ability to distinguish between motifs of different structural classes and is based on the number of times the closest matching motif returned from the database is of the same structural class as the query motif provided. As shown in Table 1, GMACS and the KFV approach recapitulates results from [12], achieving the highest average retrieval accuracy of 0.90, compared to 0.87 for the word covariance approach of MoSta, 0.87 for the STAMP platform when using PCC and ungapped local alignment, and 0.86 for the SMLR approach.

**Table 1:** **Retrieval accuracy**

	**GMACS**	**STAMP**	**MoSta**	**SMLR**
bZIP (93)	0.92	**0.94**	0.90	0.92
C2H2 (74)	**0.82**	0.76	0.76	0.77
C4 (52)	**0.98**	**0.98**	**0.98**	0.91
Homeobox (50)	**0.88**	0.82	0.82	0.85
Forkhead (49)	**0.92**	0.90	**0.92**	0.83
bHLH (37)	0.89	0.81	**0.92**	0.88
Total (355)	**0.90**	0.87	0.88	0.86

The ability of the KFV metric to distinguish different motif structural classes in the TRANSFAC benchmark dataset is compared to three alternative approaches. For each class, the highest retrieval accuracy is shown in bold. GMACS scores the overall highest average accuracy and does particularly well on the complex C2H2 zinc-finger family which causes problems for some of the other approaches.

### Motif clustering

We demonstrate the clustering performance of our algorithm on 79 motifs from the JASPAR database. This dataset comprises the 71 motifs used by [3] in their initial manual creation of FBPs, plus a further eight zinc-finger proteins, four from the DOF family, and four from the GATA family. We compare our results to those reported by the authors of STAMP [10] and MoSta [11] who both use the same dataset to benchmark their approaches. While we are primarily interested in avoiding the issue of frozen subalignments faced by tree-based approaches, we include MoSta in this comparison as it allows us to demonstrate the benefit of combining the KFV metric with a genetic-*k*-medoids approach against not only tree-based techniques, but also another alignment-free, non-tree based technique. We report the results both in terms of number of clusters defined, and the structural homogeneity of the created clusters. The ability to create structurally homogeneous clusters is an important aspect in the generation of FBPs, and as we show in the next section, GMACS performs very well in this regard, successfully identifying even distinct subtypes within several structural families. For STAMP, PCC was used with ungapped local alignment and a UPGMA guide tree (default settings). As previously described, the authors of MoSta provide their own clustering approach whereby they select and merge motifs based on their word covariance similarity metric. For this set of experiments GMACS was configured with a population of 100 which was evolved over 300 generations. The default mutation rate of 0.05 and information content threshold of 0.3 were used – this trim threshold was consistent with the same parameter setting in the STAMP algorithm.

We first provide an overall view of the solutions provided by each algorithm before examining some of the differences in detail. In total, MoSta produced 26 clusters – eleven of these are homogeneous, three are heterogeneous (containing motifs from more than one structural class), and 12 are singletons (Table 2). STAMP estimates the number of clusters at eighteen, producing nine homogeneous, seven heterogeneous, and two singleton clusters. GMACS also produces eighteen clusters, but thirteen of these are homogeneous, while the remaining five contain motifs from multiple structural families; there are no singleton clusters produced. The relatively high number of singleton clusters produced by MoSta can be attributed to the fact that their clustering approach prevents the merging of motifs if an FBP will become too heterogeneous as a result of the merge. This helps to maintain a high number of homogeneous clusters (although fewer than GMACS), but does so at the cost of an increased number of singletons. A summary of the homogeneous clusters derived by the three algorithms is shown in Additional file 1: Figure S1.

**Table 2:** **Cluster summary**

	**GMACS**	**STAMP**	**MoSta**
Homogeneous	**13**	9	11
Heterogeneous	5	7	3
Singletons	0	2	12
Total	18	18	26

Number and type of clusters automatically determined by GMACS, STAMP, and MoSta for the 79 motif JASPAR dataset originally grouped into 11 FBPs manually by Sandelin and Wasserman. GMACS achieves the highest number of homogeneous clusters while maintaining a low number of both heterogeneous clusters and singletons.

**Figure 5:**
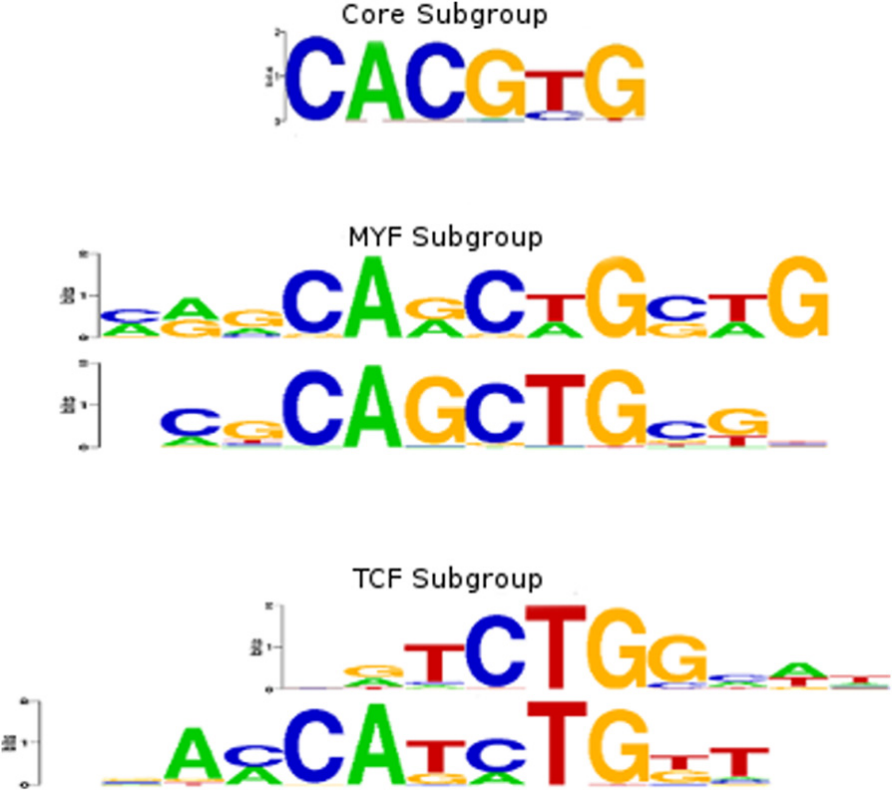
**Basic Helix-Loop-Helix family motifs.** The bHLH familty of motifs from the JASPAR dataset comprises three distinct binding subtypes. GMACS correctly classifies the motifs into these three subtypes, the core subtype containing six motifs (Arnt, AHR-ARNT, MAX, MYC-MAX, USF, n-MYC), and the MYF (MYF, NHLH1) and TCF (HAND1, TAL1) subtypes, each containing two motifs.

We begin our detailed examination of the results with the ten members of the bHLH family which form three distinct subgroups (as shown in Figure 5). STAMP creates two homogeneous clusters with six and two members respectively. Of the remaining members, one is clustered with the GATA1 zinc-finger and FOXL1 forkhead motifs, while the other is clustered with the ETS family. MoSta groups the motifs as a cluster of five and three. The larger cluster is surprisingly missing the AHR-ARNT motif which contains the strong consensus ‘CACGTG’ sequence associated with that subgroup, while the smaller cluster includes the TAL1-TCF3, NHLH1, and MYF motifs, mixing the remaining subtypes. GMACS provides the only approach to create three homogeneous clusters. The first cluster is the same six-member group created by STAMP, the second contains the NHLH1 and MYF motifs (MYF subgroup), while the final cluster groups the TAL1 and HAND1 (TCF subgroup) motifs together.

All three of the algorithms separate the four bZIP CREB subgroup motifs into a homogeneous group as well as clustering seven of the eight nuclear receptor motifs together. The remaining androgen receptor motif is classed as one of two singletons by STAMP, while GMACS clusters this motif with the two homeobox motifs, EN-1 and PAX4. It is possible that the length and complexity of the AR and PAX4 motifs play a role in this particular grouping, affecting the number of shared *k*-mers between the two.

**Figure 6:**
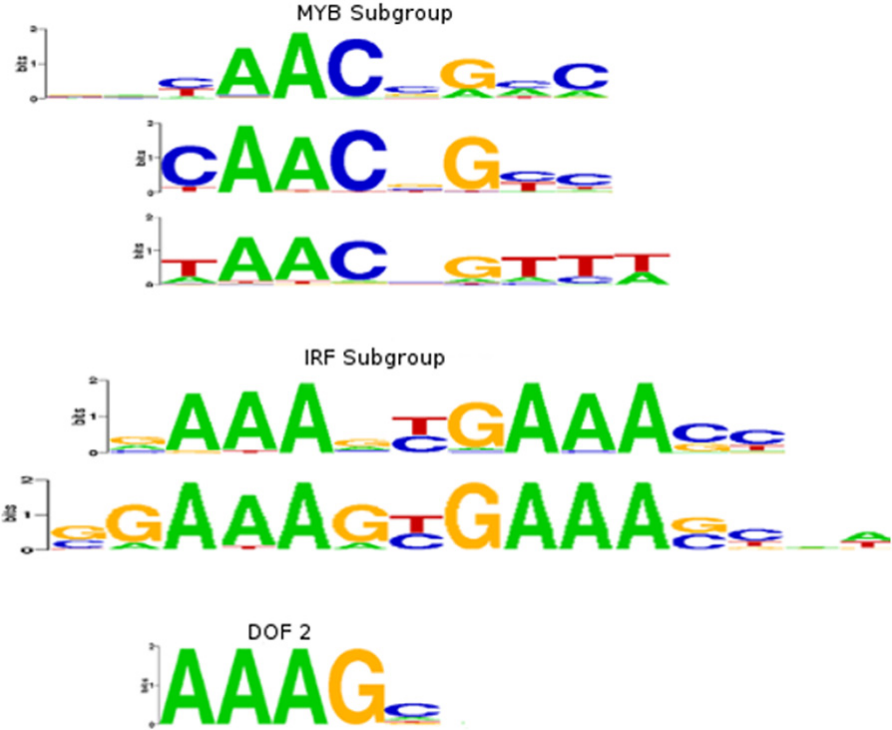
**TRP family motifs.** The TRP family of motifs comprises two binding subtypes. The first of these is the MYB group which includes three motifs (GAMYB, c-MYB, and MYB_PH3), while the second subgroup is made up of the IRF1 and IRF2 motifs (trimmed here for display purposes). Both STAMP and MoSta include a DOF family motif (an example of which is shown here) with the IRF subgroup based on a strong ‘AAAG’ signal.

The TRP group of motifs contains two distinct subfamilies. The first of these, the MYB group, are recognized by STAMP and GMACS as a homogeneous cluster of three motifs, GAMYB, c-MYB, and MYB.PH3. MoSta, on the other hand, only recognizes two of these motifs as belonging together, excluding MYB.PH3 from this cluster. The second TRP subfamily is comprised of the IRF1 and IRF2 motifs. While both STAMP and MoSta group these two motifs with the four DOF zinc-finger motifs as a single heterogeneous cluster, GMACS instead creates two homogeneous clusters. The clustering by STAMP and MoSta in this case is reasonable however, given the strong ‘AAAG’ DOF family motif signal which is easily mistaken for a submotif of the IRF family (Figure 6). Both MoSta and GMACS cluster all of the ETS and REL family motifs separately as homogeneous groups. The STAMP ETS cluster however, also includes the HAND1-TCF3 bHLH motif making this group heterogeneous, while its REL group contains the bZIP cEBP subgroup motif CHOP-cEBP. This cEBP subgroup is split into two clusters by GMACS, one is homogeneous and contains the cEBP and CHOP-cEBP motifs, while the other contains the NFIL3 and HLF cEBP motifs as well as the forkhead motif FOXC1. GMACS also incorrectly clusters a single forkhead motif, FOXL1, with the five members of the MADS family whereas STAMP and MoSta maintain the MADS group as a homogeneous cluster. This inclusion of FOXL1 with the MADS family may be explained by the shared ‘TATTTAT’ sequence.

**Figure 7:**
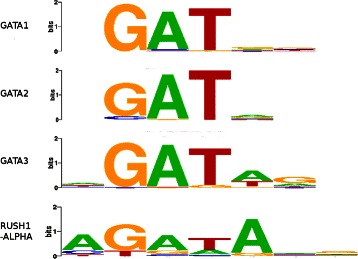
**GATA family motifs.** The four GATA motifs include the canonical ‘GATA’ consensus and are grouped together by GMACS. STAMP mis-clusters one of these with the TAL1 and FOXL1 motifs, while MoSta incorrectly classifies two of the four as singletons.

The clustering of the four highly-conserved zinc-finger GATA family motifs (Figure 7) shows considerable variation among the three algorithms. MoSta does poorly, clustering only two of the four motifs together. STAMP clusters three of the four while the remaining member is, as previously indicated, clustered with TAL1 and FOXL1. GMACS however, creates a homogeneous cluster from the four motifs.

Our final set of motifs includes members from the homeobox, HMG and forkhead families. Firstly, all three approaches cluster five of the seven homeobox motifs into one homogeneous cluster. STAMP clusters PBX with the four HMG motifs, SOX17, SOX19, SOX5 and SRY, and creates a single combined HMG/homeobox/forkhead cluster comprising both these motifs and six motifs from the forkhead family. STAMP also creates a HMG/forkhead group containing HMG-1 and FOXC1, and a HMG/homeobox group containing HMG-IY and PAX4. MoSta clusters the four HMG motifs with the six forkhead motifs as in the case of STAMP, but does not include the PBX homeobox motif. It also creates a HMG/homeobox group but in this case containing HMG-1 and EN-1. GMACS, like STAMP, clusters the PBX homeobox motif with the four HMG motifs, but as a separate cluster from the six forkhead motifs, which are instead clustered with another set of HMG motifs: HMG-IY and HMG-1.

### FBP construction and stability

**Figure 8:**
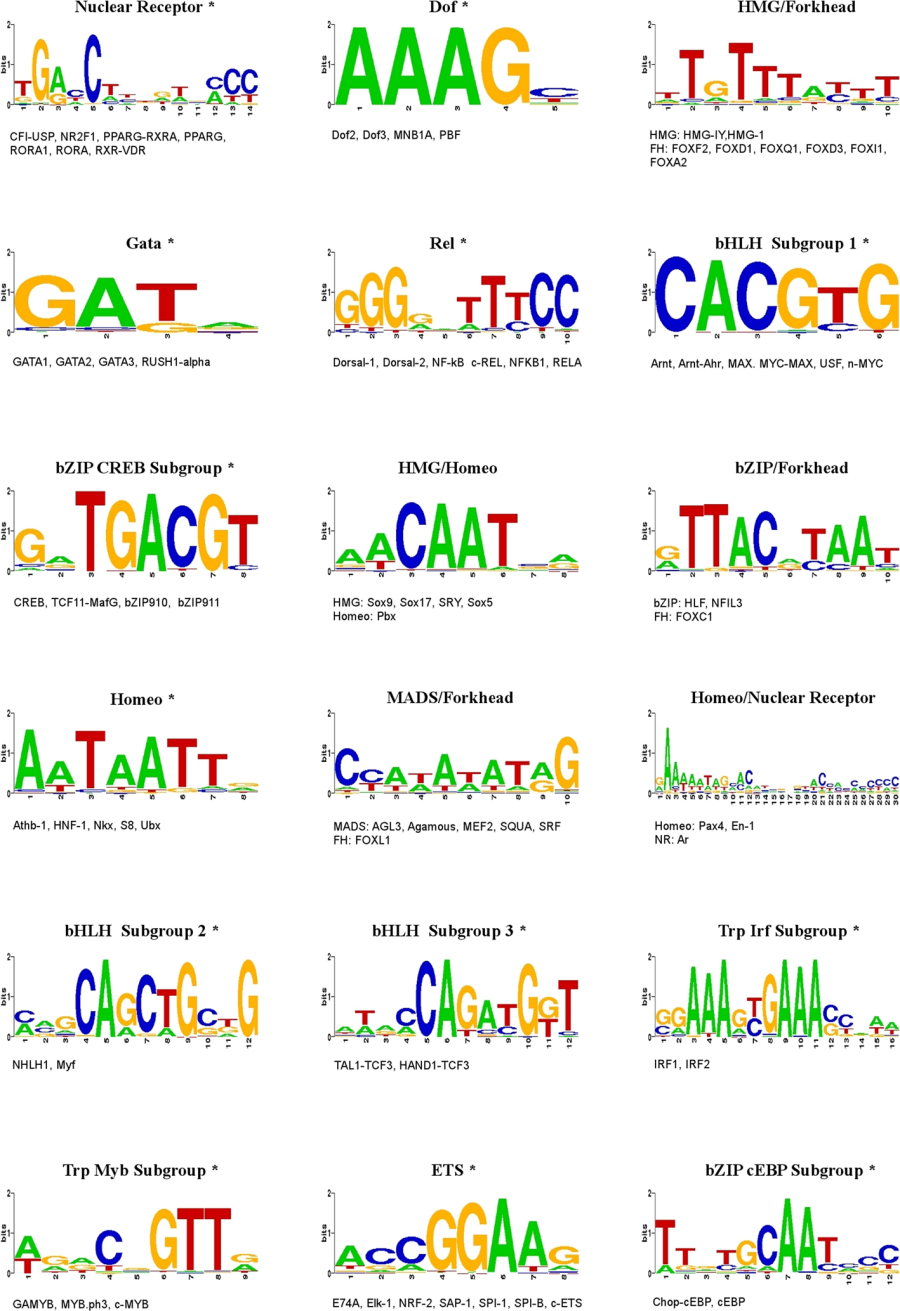
**Final cluster composition.** Cluster membership and FBPs for the eighteen clusters identified by our method in the test dataset of 79 JASPAR motifs. Clusters marked with an asterisk are structurally homogeneous.

Once we have clustered the motifs, we must generate the FBP for each of the defined clusters. This can be achieved through any of the standard multiple alignment methods, although it has previously been shown that a local Smith-Waterman alignment may be preferred for binding motifs which are typically short ungapped sequences [10]. The membership and FBPs for each of the clusters derived by GMACS for the JASPAR dataset are shown in Figure 8. In order to assess the stability of the FBPs in our solution, we perform a leave-one-out cross-validation (LOOCV) as carried out in [10,11]. GMACS achieves a LOOCV rate of 0.96 for the 79 JASPAR motifs, compared to 0.91 for STAMP. The improved classification rate for our approach is unsurprising given that more of the clusters elucidated are structurally homogeneous and therefore FBPs are less likely to be affected by the removal of any individual motif. The MoSta algorithm [11] successfully manages to re-cluster all the 67 clustered motifs to their cognate FBPs achieving a LOOCV accuracy rate of 1.0 - this result however is achieved based on the exclusion of all singleton clusters from the original solution.

### Robustness to noise

**Figure 9:**
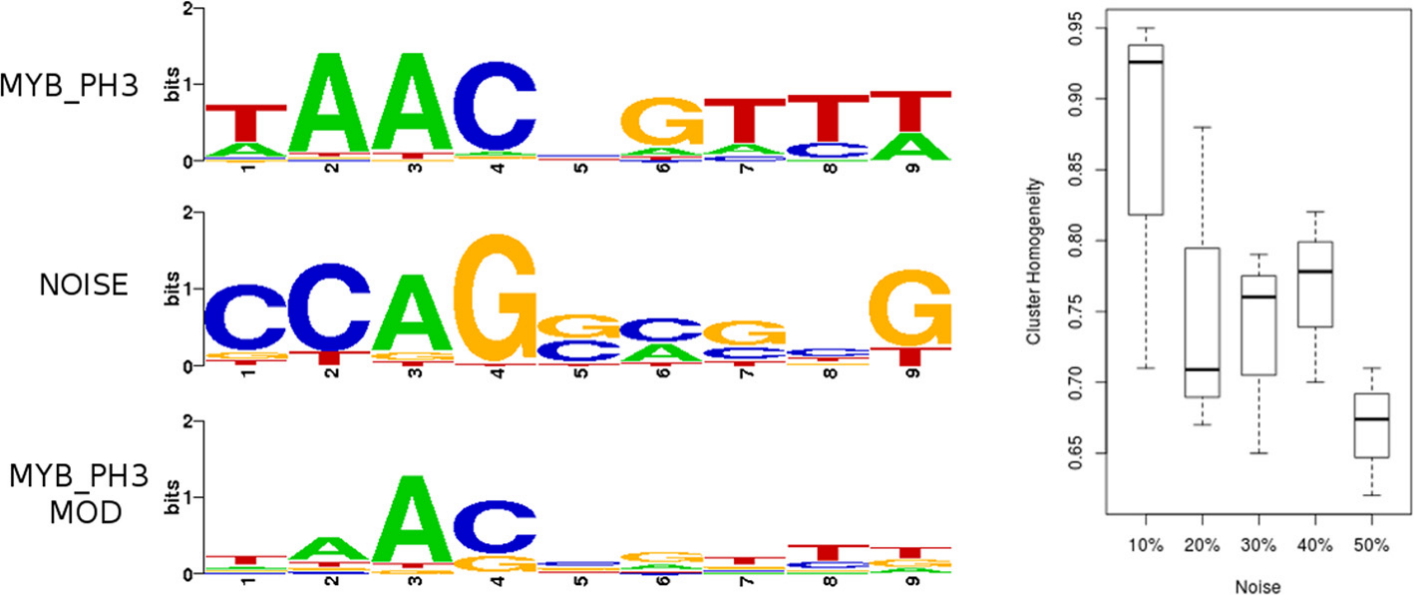
**Robustness to noise.** Left panel: The creation process for the five synthetic datasets to assess the ability of GMACS to maintain homogeneous clusters in the face of decreasing information content is depicted. The MYB_PH3 motif is combined with a motif generated by randomly sampling columns from the JASPAR database resulting in a much more degenerate motif. Right panel: The ability of GMACS to produce structurally homogeneous clusters remains robust, maintained at a level above 0.6 even when information content for each motif in the dataset has been significantly reduced.

Having benchmarked our algorithm against current state-of-the art methods, we sought to examine the boundaries of its ability to maintain homogeneous clusters in the face of low information content motifs. To that end we produced five further synthetic datasets, each comprised of the weighted combination of the original JASPAR 79 motifs and motifs generated through random sampling of columns from the entire JASPAR database. The resulting datasets comprise motifs which are 90% original motif signal and 10% random motif ‘noise’, 80% signal, 20% noise, and so on, up to equal weighting of both signal and noise. An example of this can be seen in Figure 9 (left panel), which shows the original MYB_PH3 motif, the randomly generated motif, and the modified MYB_PH3 resulting from the weighted combination of the two (0.6/0.4 respectively). Once these increasingly noisy datasets had been generated, the clustering process was repeated ten times for each set and the resulting range of cluster homogeneity at each level of random signal incorporation was examined. As shown in Figure 9 (right panel) GMACS demonstrates robustness to highly degenerate motifs, maintaining a cluster structural homogeneity score above 0.6 even when the information content has been reduced by 50%.

## Discussion

GMACS was primarily developed in order to circumvent the problem of frozen subalignments associated with tree-based FBP techniques and the results provided in the motif comparison section indicate that a genetic-*k*-medoids approach may be useful not only when compare to tree-based techniques but also to other alignment-free non-tree-based techniques as well. We must however recognize some weaknesses arising from our approach. A common concern with genetic algorithms is that they can be computationally costly, with the most compute-intensive task usually being the evaluation of the fitness function. The *k*-medoids algorithm is also computationally intensive given that the swap stage typically progresses until no further exchanges can be made to decrease the overall cost of the cluster configuration. While the complexity of the standard algorithm is *O*(*k*(*n*−*k*)^2^) (where *k* is the number of medoids and *n* is the number of objects to be clustered), we have shown that a single round of local search using the *k*-medoids as part of the fitness evaluation function is enough to greatly reduce the number of generations necessary for the GA to converge on good solutions. The silhouette component of the fitness function however requires an all-to all comparison adding a *O*(*n* ^2^) term to GMACS’ overall complexity. This indicates that while increases in other parameters such as number of generations and population size will invariably increase the runtime, the rate-limiting step will inevitably be the number of motifs in the input dataset. For the relatively small test dataset of 79 JASPAR motifs however, setting a population size of 100 and evolving for 300 generations, the average time to completion on a 2.0 GHz Intel Core i7 2630QM processor calculated over 100 runs was 3.99 seconds. This is in comparison to 6.87 seconds for STAMP and 111.19 seconds for MoSta (Additional file 2: Figure S2). Genetic algorithms are however amenable to parallelization, and future work on decreasing runtime will focus on this aspect of development.

While GMACS is designed to seek a global rather than a local minimum, like all GAs, it is a stochastic algorithm and there are therefore no guarantees that it will in fact achieve this goal. In order to test its convergence properties, we ran the test dataset of 79 JASPAR motifs 10,000 times to ascertain the number of times which the algorithm would converge on any particular solution. The 18-cluster solution (*K*=18) which we have described above accounts for ∼90.5% of the solutions returned and has a fitness value of 0.469. The second most common solution, accounting for a further 5% of the returned cluster configurations, is a 19-cluster solution with a fitness value of 0.465. In this solution, the FOXC1 motif is classified as a singleton, resulting in the NFIL3 and HLF cEBP motifs becoming a homogeneous cluster. The third most common solution occurs in ∼1.2% of the runs and is another 19-cluster solution, also involving a FOXC1 singleton. This time however, the forkhead group becomes homogeneous and the two HMG motifs from the previous HMG / forkhead group are re-clustered elsewhere. The fitness for this third solution is in fact slightly higher (0.471) than that of the *K*=18 solution, raising several important points. Firstly, it illustrates the fact that the GA will quickly converge on good but not necessarily optimal solutions. This result also points to the difficulty, not only for GAs but also for most algorithms operating in complex problem domains, of appropriate parameter selection. The trim threshold, mutation rate, population size, and number of generations, for example, will all play a role in the type of solutions returned. Finally, the higher fitness of the *K*=19 solution provides an indication that a modified or alternative fitness function could help to move the GA towards these less commonly explored regions of the solution space. There are many cluster quality metrics which might be used as part of the fitness function as well as many possible implementations of the genetic operators – future work will focus on exploring these possible combinations in an attempt to further optimize the clustering process.

Our results also confirm the *K*-mer Frequency Vector as a suitable metric for general motif similarity. However, while this approach achieves the best average retrieval accuracy overall, it seems clear that each of the metrics examined are more sensitive to certain structural classes than others. An ensemble approach or weighted combination of metrics may therefore provide a more optimal way to ascertain a motif’s structural class.

## Conclusion

While being cognizant of the issues raised in the previous section, we posit that our algorithm is a useful complementary technique to current standard approaches. The most common clustering solution provided by GMACS for the benchmark clustering dataset is both consistent and biologically meaningful, comprising a larger number of structurally homogeneous clusters than either STAMP or MoSta, without requiring a large number of singleton clusters to achieve this. As well as being applicable to other motif clustering problems, our algorithm is easily reconfigurable for other classes of general clustering problems, making it particularly attractive to researchers wishing to test the robustness of results returned by conventional tree-based approaches.

## Availability and requirements

**Project name:** GMACS **Project home page:** http://goldenlab.einstein.yu.edu/projects/gmacs **Operating system(s):** Platform independent **Programming language:** C**Other requirements:** none**License:** GMACS is freely available for download and use under the GNU GPL

## Additional files

Additional file 1
**Figure S1.** Homogeneous cluster overlap. This figure provides a Venn diagram summary of the overlap in terms of homogeneous clusters created by the three algorithms tested.

Additional file 2
**Figure S2.** Algorithm runtimes. Runtime boxplots are shown for the three algorithms across 100 replicates of the JASPAR dataset.

## References

[1] Fogel GB, Weekes DG, Varga G, Dow ER, Craven AM, Harlow HB (2005). (2005) A statistical analysis of the TRANSFAC database. Biosystems.

[2] Stormo GD (2000). DNA binding sites: representation and discovery. Bioinformatics.

[3] Sandelin A, Wasserman WW (2004). Constrained binding site diversity within families of transcription factors enhances pattern discovery bioinformatics. J Mol Biol..

[4] Sandelin A, Alkema W, Engström P, Wasserman WW, Lenhard B (2004). JASPAR: an open-access database for eukaryotic transcription factor binding profiles. Nucleic Acids Res..

[5] Mahony S, Golden A, Smith TJ, Benos PV (2005). Improved detection of DNA motifs using a self-organized clustering of familial binding profiles. Bioinformatics.

[6] Xing EP, Karp RM (2004). MotifPrototyper: A Bayesian profile model for motif families. Proc Natl Acad Sci USA.

[7] Narlikar L, Hartemink AJ (2006). Sequence features of DNA binding sites reveal structural class of associated transcription factor. Bioinformatics.

[8] Kielbasa SM, Gonze D, Herzel H (2005). Measuring similarities between transcription factor binding sites. BMC Bioinformatics.

[9] Schones DE, Sumazin P, Zhang MQ (2004). Similarity of position frequency matrices for transcription factor binding sites. Bioinformatics.

[10] Mahony S, Auron PE, Benos PV (2007). DNA familial binding profiles made easy: comparison of various motif alignment and clustering strategies. PLoS Comput Biol..

[11] Pape UJ, Rahmann S, Vingron M (2008). Natural similarity measures between position frequency matrices with an application to clustering. Bioinformatics.

[12] Xu M, Su Z (2010). A novel alignment-free method for comparing transcription factor binding site motifs. PLoS ONE.

[13] Mahony S, Benos PV (2007). STAMP: a web tool for exploring DNA-binding motif similarities. Nucleic Acids Res.

[14] Smith TF, Waterman MS (1981). Identification of common molecular subsequences. J Mol Biol..

[15] Needleman SB, Wunsch CD (1970). A general method applicable to the search for similarities in the amino acid sequence of two proteins. J Mol Biol..

[16] Sokal RR, Michener CD (1958). A statistical method for evaluating systematic relationships. Univ Kans Sci Bull..

[17] Barton GJ, Sternberg MJ (1987). A strategy for the rapid multiple alignment of protein sequences. Confidence levels from tertiary structure comparisons. J Mol Biol..

[18] Lloyd SP (1982). Least squares quantization in PCM. IEEE T Inform Theory..

[19] Kaufman L, Rousseeuw PJ (1990). Finding groups in data: an introduction to cluster analysis.

[20] Fraser AS (1957). Simulation of genetic systems by automatic digital computers I. Introduction. Aust J Biol Sci..

[21] Holland JH (1975). Adaptation in natural and artificial Systems.

[22] Goldberg DE (1989). Genetic algorithms in search, optimisation and machine learning.

[23] Notredame C, Higgins DG (1996). SAGA: sequence alignment by genetic algorithm. Nucleic Acis Res..

[24] Notredame C, O’Brien EA, Higgins DG (1997). RAGA: RNA sequence alignment by genetic algorithm. Nucleic Acids Res..

[25] Wei Z, Jensen ST (2006). GAME: detecting cis-regulatory elements using a genetic algorithm. Bioinformatics.

[26] Liu FFM, Tsai JJP, Chen RM, Chen SN, Shih SH. FMGA: finding motifs by genetic algorithm. In: Fourth IEEE symposium on Bioinformatics and Bioengineering (BIBE2004). IEEE2004. p. 459–66.

[27] Bohlin J, Skjerve E, Ussery DW (2008). Investigations of oligonucleotide usage variance within and between prokaryotes. PloS Comput Biol..

[28] Tibshirani R, Walther G, Hastie T (2001). Estimating the number of data clusters via the Gap statistic. J Roy Stat Soc B..

[29] Calinski T, Harabasz J (1974). A dendrite method for cluster analysis. Commun Stat..

[30] Rousseeuw PJ (1987). Silhouettes: a graphical aid to the interpretation and validation of cluster analysis. Comput Appl Math..

[31] Matys V, Fricke E, Geffers R, Gößling E, Haubrock M, Hehl R (2003). TRANSFAC: transcriptional regulation, from patterns to profiles. Nucleic Acids Res..

