# Supplementary figures and images for: Alignment-free clustering of transcription factor binding motifs using a genetic-k-medoids approach
Source: BMC Bioinformatics. 2015 Jan 28;16:22. doi: 10.1186/s12859-015-0450-2 (PMC4384390; doi:10.1186/s12859-015-0450-2)

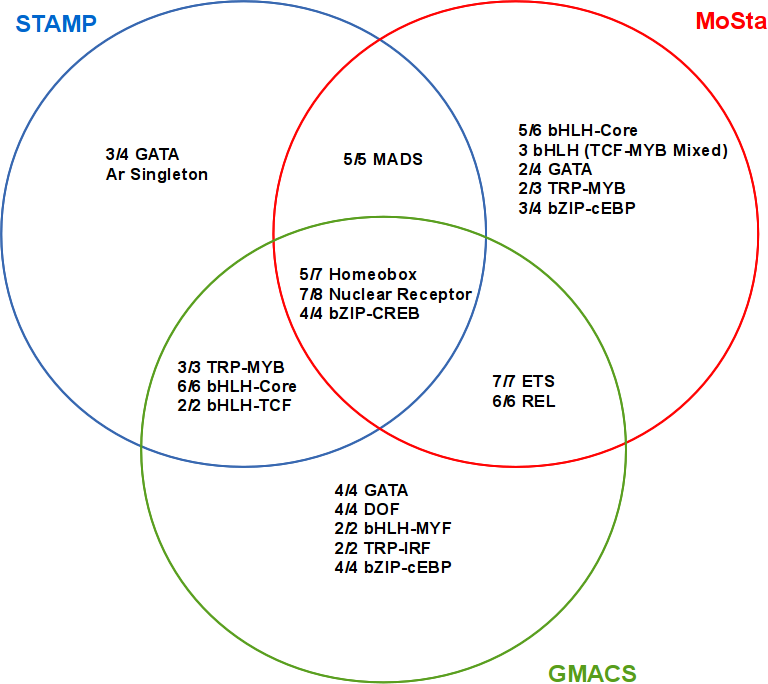

Supplement: Additional file 1 — Figure S1. Homogeneous cluster overlap. This figure provides a Venn diagram summary of the overlap in terms of homogeneous clusters created by the three algorithms tested. [file 12859_2015_450_MOESM1_ESM.tiff]
